# Source-to-Sink Translocation of Carbon and Nitrogen Is Regulated by Fertilization and Plant Population in Maize-Pea Intercropping

**DOI:** 10.3389/fpls.2019.00891

**Published:** 2019-07-09

**Authors:** Yanhua Zhao, Zhilong Fan, Falong Hu, Wen Yin, Cai Zhao, Aizhong Yu, Qiang Chai

**Affiliations:** ^1^College of Resources and Environmental Sciences, Gansu Agricultural University, Lanzhou, China; ^2^Gansu Provincial Key Laboratory of Aridland Crop Science, Lanzhou, China; ^3^College of Agronomy, Gansu Agricultural University, Lanzhou, China

**Keywords:** carbon and nitrogen accumulation, carbon and nitrogen translocation, intercropping, plant density, inter-plant competition

## Abstract

Translocation of carbon (C) and nitrogen (N) from vegetative tissues to the grain sinks is critical for grain yield (GY). However, it is unclear how these processes respond to crop management practices when two crops are planted in relay-planting system. In this study, we characterized the C and N accumulation and translocation and their effects on yield formation in a pea (*Pisum sativum* L.)-maize (*Zea mays* L.) relay-planting system under different levels of source availabilities. Field experiment was conducted at Wuwei, northwest China, in 2012, 2013, and 2014. Two N fertilizer rates (low – N0 and high – N1) and three maize plant densities (low – D1, medium – D2, and high – D3) were designed to create the different levels of source availabilities. During the co-growth period, the rate of C accumulation in intercropped maize was 7.4–10.8%, 13.8–22.9%, and 13.5–32.0% lower than those in monoculture maize, respectively, under the D1, D2, and D3 treatments; however, after pea harvest, these values were 1.1–23.7%, 33.5–78.9%, and 36.8–123.7% greater than those in monoculture maize. At maturity, intercropped maize accumulated 11.4, 11.5, and 19.4% more N than monoculture maize, respectively, under the D1, D2, and D3 treatments. Compared to the monoculture crops, intercropped pea increased C accumulation in stems by 40.3% with N-application and by 19.5% without N application; intercropping maize increased these values by 16 and 11%, respectively. Overall, increasing N fertilization improved the rates of C and N remobilization from the vegetative tissues to the grain sinks across the different density treatments. In intercropped maize, the stems contributed 22, 33, and 44% more photosynthate to the grain sinks than the leaves, respectively, under the D1, D2, and D3 treatments. Quantitative assessments showed that the enhanced C and N remobilization due to high N fertilization and high plant density led to an increase of GY in the intercropping system by 35% compared with monoculture. We conclude that the enhanced productivity in maize-pea intercropping is a function of the source availability which is regulated by plant density and N fertilization.

## Introduction

Feeding the world with a growing population is an enormous challenge (Tanton et al., 2003; Fedoroff et al., 2010; Larson, 2013), and the challenge is exacerbated in highly populated countries such as China and India where the small farmable land area per capita is rapidly shrinking due to urban construction and economic expansion in other sectors (such as Highways construction) that compete for land with agriculture (Godfray et al., 2010; Cumming et al., 2014). Conventional high-input farming systems, used for decades in areas with high food demands, are shown to have significant negative impacts on the environment as the high input of synthetic fertilizers and pesticides increases greenhouse gas emissions that contribute to climate change (Chen et al., 2014c; Cui et al., 2018). Also, excessive use of synthetic fertilizers for years may cause soil acidification (Zhang et al., 2015) and increase the risk of soil pollution (Chen et al., 2014b). In line with the United Nation’s “Climate-Smart Agriculture” Action Plans (FAO/UNESCO, 2018), intensifying cropping systems has been considered a key strategy to grow more food on the existing land (Tilman et al., 2011). Intercropping, a system enabling the simultaneous production of multiple crops on the same area of land, is a proven advancement for crop intensification (Li et al., 2016), and is considered a new “Green Revolution” (Martin-Guay et al., 2018) in a way to satisfy the nutritional needs of a growing population whilst limiting environmental repercussions.

Intercropping has been reported to have significant yield advantages over the corresponding sole cropping (Chai et al., 2014; Hu et al., 2016, 2017) due to more efficient use of available resources (Franco et al., 2018), such as soil water, and nutrients (Mu et al., 2013; Chen et al., 2018). Also, the coordination of competition and complementation between intercrops play an important role in the yield advantage (Wasaya et al., 2013; Tanwar et al., 2014). During the co-growth period (i.e., the period when the two crops grow together), the growth of one crop influences the performance of the accompanying crop, while some niche differentiation between intercrops usually occurs in the context of space and time (Brooker et al., 2015; Zhang W. P. et al., 2016). In mixing cropping, asymmetrical interspecific competition often occurs (Klimek-Kopyra et al., 2017a) and the magnitude of the competition varies with crop species and agronomic practices (Klimek-Kopyra et al., 2018) and weather conditions (Klimek-Kopyra et al., 2017b). The degree of the interspecific competition for resources may be reduced through a possible compensatory effect between the intercrops (Chen et al., 2014a; Franco et al., 2018). For example, intercropping of a cool-season, earlier-maturing crop with a warm-season, later-maturing crop can lead to a “sharing” of available soil nutrients between the intercrops (Li et al., 2014). Also, a large compensatory effect on the later-maturing crop may occur (Chen et al., 2018), as the later-maturing crop accelerates its growth with all the available resources after the harvest of the earlier-maturing intercrop, leading to a full recovery from the inhibited growth encountered during the co-growth period (Li et al., 2014; Chen et al., 2018).

Key processes in the formation of crop yield are carbon (C) and nitrogen (N) accumulation in plant tissues (Xie et al., 2014; Hossain et al., 2018) and the translocation of the photosynthates from vegetative tissues to the grain sink (Anbessa et al., 2007; Xie et al., 2015). The outcome of these processes is largely reflected by nutrients available to the crop (Gan et al., 2010) and the uptake capacity of the host plants (Niu et al., 1998; Wang et al., 2008). In intercropping systems, the accumulation and translocation of C and N of the interspecies during the co-growth period as well as during the postharvest of the early-maturing crop can have a significant impact on the outcome of intercrop productivity (Yin et al., 2016).

In conventional monoculture systems, high availability of C sources leads to higher C accumulation in the sink (van Roekel and Coulter, 2011; Antonietta et al., 2014). However, little information is available about the C and N accumulation and translocation in response to the availability of C and N sources in intercropping systems. To maximize the benefits of intercropping, it is essential to understand the source-sink relationship for both C and N under different management strategies. The translocation of the C and N sources to the sink may affect the competitiveness between intercrops and the complementary effects from one intercrop to the other.

Therefore, the objectives of the study were to determine (i) the source-sink relationship under the different levels of C and N source availability in maize (*Z. mays* L.) – pea (*P. sativum* L.) intercropping, a typical cereal-legume intercropping pattern adapted in many arid and semiarid areas; and (ii) evaluate the interspecific competition and complementation during the co-growth period as well as postharvest the early-maturing pea in response to the different levels of source availability. Two levels of N fertilizer and three maize plant densities are designed to create the levels of C and N source availability. We hypothesized that the source-to-sink translocation in C and N is a function of the N fertilizer and plant density in intercropping systems as these two factors likely involve in the regulation of source availability.

## Materials and Methods

### Experimental Site

The field experiment was carried out at the Oasis Agricultural Experimental Station (37°30′N, 103°5′E; 1776 m a.s.l.) of Gansu Agricultural University, Wuwei, northwestern China, in 2012, 2013, and 2014. The station is in the eastern part of Hexi Corridor, with a typical oasis climate. The long-term (1960–2009) average daily total global radiation is 15.53 MJ m^–2^ d^–1^, mean annual temperature is 7.2°C with accumulated temperature above 0°C > 3513°C, and the frost-free period 156 days. Annual average precipitation is 155 mm, with the large proportion of rainfall occurring in July through September, and annual evaporation is about 2400 mm (Chai et al., 2016). In the present study, we recorded weather data using a Farmland Microclimate Automatic Monitoring System (Hangzhou, China). Sunshine hours during the study years of 2012, 2013, and 2014 were 2926, 3012, and 2523 h, respectively; average air temperature was 6.8, 7.9, and 7.2°C; frost-free period was 167, 141, and 158 days; and annual precipitation was 124.3, 123.2, and 278.3 mm, respectively. These weather variables in the study years were comparably similar to the long-term averages. The soil at the experimental site is an Aridisol (FAO/UNESCO, 1988), with sandy loam texture. At the beginning of the experiment, total nitrogen (N), NH_4_^+^-N, and NO_3_^–^-N in the 0–30 cm soil layer were 0.94 g kg^–1^, 1.78 mg kg^–1^, and 12.5 mg kg^–1^, respectively. Soil organic matter was 14.3 g kg^–1^.

### Experimental Design and Plot Management

In each of the three study years, the experiment included three factors with 14 treatments arranged in a randomized, complete block design with three replicates. The first factor was two cropping systems: maize-pea intercropping and corresponding sole plantings; the second factor was two N rates: 0 kg N ha^–1^ (N0) and 450 kg N ha^–1^ for maize (N1), and 0 kg N ha^–1^ (N0) and 135 kg N ha^–1^ for pea (N1); and the third factor was three plant densities in maize: low (73,600 plants ha^–1^ in sole planting, and 42,600 plants ha^–1^ in intercropped maize), medium (85,900 and 49,700 plants ha^–1^, respectively) and high (98,200 and 56,900 plants ha^–1^, respectively). The cropping systems were treated as the main plot, and the N rates and plant density as the subplots. Row spacing was the same for the intercropped maize and the sole maize. The rate of fertilizer applied was the same for the intercropped maize and the sole maize per unit area. Urea (46-0-0 of N-P_2_O_5_-K_2_O) fertilizer was used with 20% of the total N (i.e., 90 kg N ha^–1^) as base N applied between rows and incorporated into the soil 30 cm deep using a shallow rotary tillage 2 days prior to sowing. The remaining N was top-dressed to maize at the V6 (rapid stem elongation), V9 (9 to 10 leaf), and VT (tasseling) stages (Ransom, 2013), with 10, 40, and 30% of the total amount of N, consecutively. For pea, 90 kg N ha^–1^ was applied at sowing as base fertilizer, and the remaining N was applied as topdressing at early flowing. For N topdressing in maize, a hole of 3 cm diameter was made to 10 cm deep 4–5 cm far from each maize plant, N fertilizer was applied to the hole, and the hole was filled with the same soil. The N topdressing in pea was implemented using broadcasting. All plots received the same amount of P fertilizer at 150 kg P_2_O_5_ ha^–1^ broadcasted at sowing.

Pea (cv. MZ-1) was planted on 1, 2, and 1 April in 2012, 2013, and 2014, respectively, using a plot seeder, and was harvested on 5, 7. and 8 July in the three respective years. Maize (cv. Xian-yu 335) was sown on 21, 22, and 25 April using an in-house built planter and was harvested on 22, 25, and 29 September, in the three respective years. Each plot was 45.6 m^2^ (5.7 × 8 m) in size. A ridge of 50 cm wide by 30 cm high was built between two adjacent plots to reduce potential water movement between plots. Plastic films were applied to maize strips at sowing to optimize seedling establishment (Gan et al., 2013). In the maize/pea intercropping system, the maize strip was 110 cm in width with 3 rows and 40 cm row spacing; the pea strip was 80 cm in width with 4 rows and 20 cm row spacing. Each plot had three sets of maize-pea strips. In a plot, intercropped maize occupied 58% of the area and intercropped pea occupied the remaining 42%. For monoculture maize, each plot had 5 strips with 3 rows per strip, and the row space was 40 cm, the same as that in intercropping system under each density treatment. For monoculture pea, there were 28 rows in each plot with a row space of 20 cm.

Irrigation was applied using flood method to all plots at a total amount of 350 mm for pea and 555 for maize. Of which, 120 mm was applied before soil freezing the previous fall, and the remaining amounts were applied as follow: 75 mm at pea seedling (prior to maize was sown), 90 mm at pea early flowering [coincident to the V6-V7 stage for maize (Ransom, 2013)], and 75 mm at pea podding (V8-V9 for maize). Maize received additional irrigation of 90 mm each at the V14-V15, VT11, and R1 stage.

### Data Collection and Indices Calculation

#### Plant Sampling

Maize and pea plants were sampled at a 15 day interval starting 20 days after pea seedling emergence (DAS) until pea harvest. Maize samplings were continued at a 20 day interval after pea harvest until maize harvest. At each sampling, 10 individual pea plants and 3 individual maize plants were taken randomly from each plot for the determination of above-ground dry matter. The plant samples were oven-dried at 105°C for 30 min and continued to dry at 80°C to a constant weight and weighed for dry matter. At the flowering and maturing samplings, each sampled pea plant was separated into leaf, stem and pod, and each maize plant was separated into leaf, stem and ear per plant. Dried grain and each part of the straw sample were milled, sieved through 1 mm screen size, and analyzed for C and N concentrations using a high-induction furnace C and N analyzer (Elementar vario MACRO cube, Hanau, Hessen, Germany). The aboveground C accumulation (kg ha^–1^) was determined as the product of C concentration and dry weight, so does for plant N accumulation. All the plants in each plot were harvested at full maturity, cleaned, air-dried, and weighed for grain yields (GYs).

#### Determination of Carbon and Nitrogen Translocation

The C and N accumulated in the vegetative tissues are believed to contribute to the developing grains during the reproductive period. We used two terms (translocation, and translocation efficiency) to describe the characteristics of C and N translocation from the vegetable tissues to the grain sink. The term “translocation” presents the quantity (kg ha^–1^) of translocated materials on an absolute value basis, whereas the term “translocation efficiency” presents percent peak value (i.e., the maximum amount of biomass) that was translocated from vegetative tissues to grain sinks.

The relative translocation of C from the vegetative tissues to the grain was determined as follows:

(1) $${\mathrm{Wi}}_{t}={\mathrm{Wi}}_{\max}-{\mathrm{Wi}}_{\mathrm{mat}}$$

where Wi_t_ is the amount of C translocated from the vegetative tissues to the grain, Wi_max_ is the maximum amount of C accumulated in a particular tissue during the growth period, and Wi_mat_ is the amount of C measured at grain maturity. The Wi_max_ was found at the late-flowering stage in pea and at the R0 stage in maize, whereas the Wi_mat_ was at grain maturity for both pea and maize.

Carbon translocation efficiency was determined as percent C exported from the vegetative tissues relative to the maximum amount of C accumulated in the tissues as follows:

(2) $$\mathrm{TE}=\frac{{\mathrm{Wi}}_{t}}{{\mathrm{Wi}}_{\max}}\times 100\%$$

where TE is the translocation efficiency; Wi_t_ and Wi_max_ are defined above.

The two equations described above were also used to quantify N translocation and N translocation efficiency, in the same way as calculated for the carbon.

#### Determination of Compensatory Effect

In the maize-pea intercropping system, the intercropped maize plants often grow disadvantageously during the co-growth period with intercropped pea, largely because of the later sowing of maize. However, after the earlier-maturing intercropped pea is harvested, the later-maturing intercropped maize may receive a certain degree of “compensation” from the intercropped pea. This phenomenon is called a “compensatory effect” (Chen et al., 2014a; Yin et al., 2017). It is an indication of the growth recovery the intercropped maize may achieve after intercropped pea is harvest. In the study, we determined the compensatory effect (CE) by comparing the relative crop growth rate (CGR) (kg ha^–1^ d ^–1^) of the intercropped maize with that of sole maize during the period from pea harvest to maize maturity, as follow:

(3) CGR=(W-2W)1/(t-2t)1;

(4) CE=CGR/IntCGRsole

where W_2_ and W_1_ represent dry matter accumulation of maize plants at the two consecutive measuring times t_2_ and t_1_ after pea harvest; CGR_Int._ and CGR_sole_ are CGR of the intercropped maize and sole maize, respectively. The CE value greater than 1.0 indicates that intercropped maize has a positive “compensatory effect” from the accompanying pea; an CE value smaller than 1.0 indicates that intercropped maize has a negative effect; and an CE value equals 1.0 meaning no “compensatory effect.”

#### Determination of Land Equivalent Ratio, Yield, and Harvest Index

Land equivalent ratio (LER) is calculated as follows:

(5) LER=LER+ALER=B(Y/intAY)monoA+(Y/intBY)monoB

Where Y_intA_ and Y _intB_ are the GYs of intercrop A and intercrop B, and Y_monoA_ and Y_monoB_ are the yields of corresponding monoculture A and monoculture B. LER_A_ and LER_B_ represent the LER of the intercrop A and intercrop B, respectively. The LER value greater than 1.0 means a yield advantage of the intercrops over the corresponding monoculture crop (Willey, 1979).

Grain yield per unit area was determined for each intercrop based on the planted areas in each plot. Harvest index (HI) was calculated by dividing the GY by aboveground biomass yield (BY) per unit area, as HI = GY/BY.

### Statistic Analysis

Data were subject to ANOVA for a standard split-plot design using SPSS program (SPSS software, 17.0, SPSS Institute Ltd., United States) with the cropping systems as the main plot, and the N rates and plant density as the subplots. Significant differences between treatments were determined with LSD at the 0.05 probability level. Cropping pattern, plant density, and N rate were regarded as fixed effects and replication as random effects. For the variables following a similar trend of treatment effects among the study years, the data of the three years were pooled together in the analysis and the 3-year means were presented, such as the variables C and N accumulation, C and N translocation, and plant growth rate. For variables where the treatment effects differed among years, the effect was determined separately for each of the study years, such as the spatial distribution of dry matter accumulation in plant tissues, GY, and HI. Further, plant density × fertilizer rate interaction was determined for each variable in these analyses. When density × N rate interaction was significant, the density effect was discussed at each N rate or N rate effect was discussed at each density; when density × N rate interaction was not significant, the mean effect among treatments was discussed.

## Results

### C and N Accumulation in Pea and Maize Plants

#### C Accumulation

**FIGURE 1:**
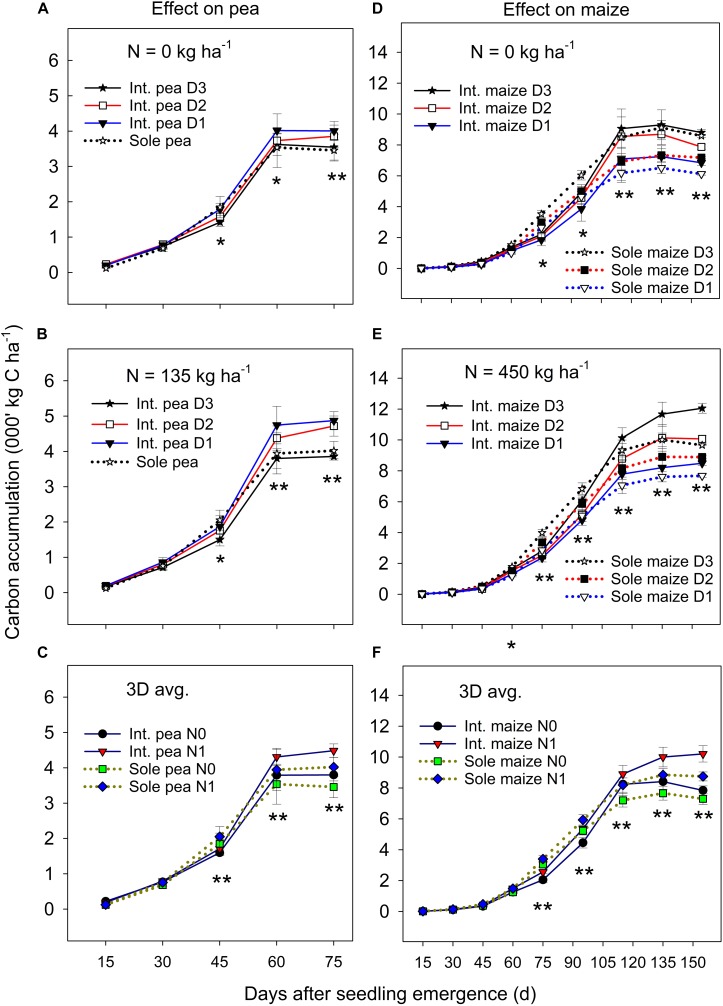
Carbon (C) accumulation in pea **(A–C)** and maize **(D–F)** plants grown in the intercropping and sole cropping systems under the two N fertilizer levels and three maize plant densities (D1, D2, and D3), averaged across three study years (*n* = 9, i.e., 3 replicates each year × 3 years). ^*^, and ^∗∗^ at each sampling date represent significant differences at *P* < 0.05 and *P* < 0.01, respectively. The line bar at each data point is standard error (*n* = 9).

The ANOVA revealed that there was no significant year by treatment interactions for C accumulation and translocation, thus, 3-year means were presented. Overall, the C accumulation in the intercropped pea and sole pea followed a similar pattern (Figures 1A–C); C accumulation increased rapidly from 15 days after seedling emergence (DAS) to 60 DAS and then leveled off or declined to plant maturity. Intercropped pea under the D1 and D2 maize densities accumulated significantly more C than intercropped pea under D3 and sole pea at DAS 60 and DAS 75 under zero N (Figure 1A) and *N* = 135 kg N ha^–1^ treatments (Figure 1B). There was a significant N rate × cropping system × sampling date interaction in affecting C accumulation in pea (Figure 1C). At DAS = 45, 60, and 75, pea plants with the *N* rate = 135 kg ha^–1^ accumulated significantly higher amounts of C than pea under the zero N treatment for both intercropping and sole pea cropping. However, such a difference between the two cropping systems or the two N levels did not show before DAS = 45 (Figure 1C).

Maize plant density affected the C accumulation of both intercropped and monoculture maize significantly after jointing (i.e., DAS = 75) and the magnitude of the effect varied with N fertilization (Figures 1D,E). Maize increased C accumulation significantly with the increase of plant density from D1 to D3 in both intercropping and monoculture systems from DAS 75 to 155. Between the two cropping systems, monoculture maize had a greater C accumulation than intercropped maize from DAS 75 to 90, but the opposite was true from DAS 115 to 155 during which intercropped maize accumulated significantly greater C than monoculture maize. There was a slow C accumulation period before DAS 60, followed by a rapid accumulation from DAS 60 to 115, and then leveled off or declined to maize maturity. During the slow C accumulation period (i.e., before DAS 60), either plant density or cropping systems had an impact on C accumulation.

On average, the C accumulation of intercropped maize was 7.4–10.8%, 13.8–22.9%, and 13.5–32.0% lower than that of monoculture maize before pea harvest (DAS 75) at the D1, D2, and D3 treatments, respectively, however, these values were 1.1–23.7%, 33.5–78.9%, and 36.8–123.7% higher than that of monoculture maize after pea harvest. With N fertilization, intercropped maize increased total C accumulation more than monoculture maize, especially after DAS 115 (Figure 1F). Even though the C accumulation of intercropped maize was inhibited during the co-growth period, a strong compensatory effect on maize growth after pea harvest occurred which offset the disadvantages encountered during the co-growth period.

#### N Accumulation

**FIGURE 2:**
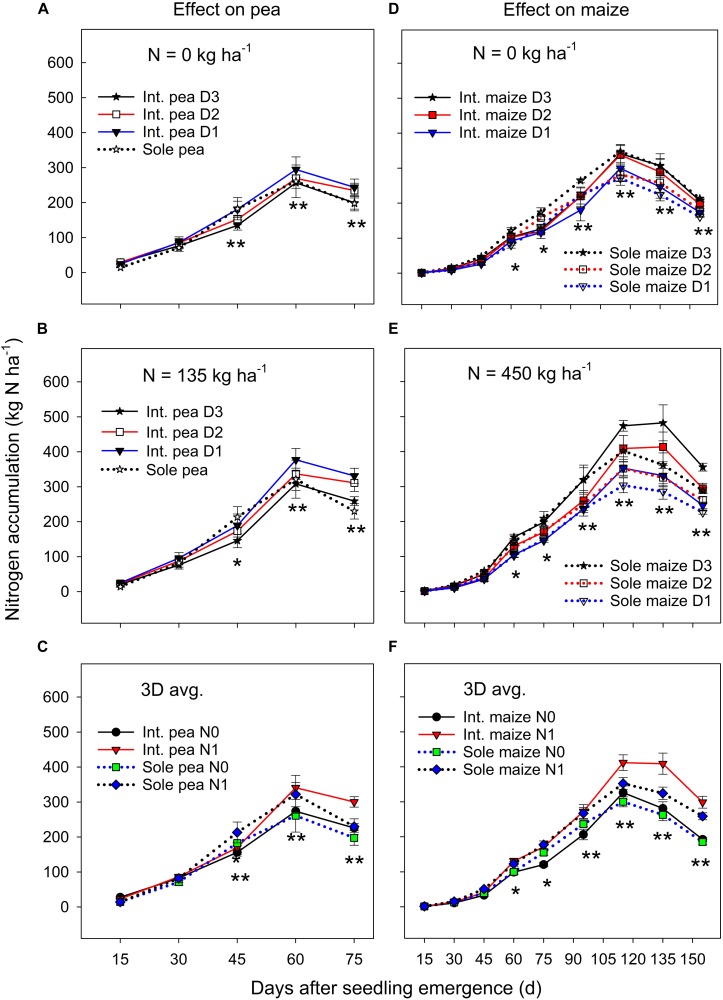
Nitrogen (N) accumulation in pea **(A–C)** and maize **(D–F)** plants grown in the intercropping and sole cropping systems under the two N fertilizer levels and three maize plant densities (D1, D2, and D3), averaged across three study years (*n* = 9, i.e., 3 replicates each year × 3 years). ^*^, and ^∗∗^ at each sampling date represent significant differences at *P* < 0.05 and *P* < 0.01, respectively. The line bar at each data point is standard error (*n* = 9).

There was no significant year by treatment interactions for N accumulation and translocation, thus, 3-year means were presented. Sole pea and intercropped pea had a similar N accumulation during the early growth period, but sole pea increased N accumulation significantly more than intercropped pea from DAS 30 to 45 (Figures 2A,B). Thereafter (i.e., from DAS 45), to pea maturity, intercropped pea accumulated significantly more N than sole pea. Pea plants in both monoculture and intercropping systems had a similar N accumulation pattern; it increased rapidly until DAS 60 and then declined to plant maturity (Figures 2A–C). Averaged across three plant densities, N fertilization had little or no effect on N accumulation in pea before DAS 45, but thereafter fertilized pea accumulated significantly greater amount of N than pea without N fertilization (Figure 2C).

There was a significant cropping system × maize plant density × sampling date interaction for N accumulation under zero N fertilizer treatment (Figure 2D), where the treatments did not differ in the N accumulation before DAS 45, but sole maize had a significantly greater N accumulation than intercropped maize from DAS 45 to 90, and thereafter the opposite was true in which intercropped maize accumulated significantly more N than sole maize until maize maturity. However, these interactions were altered by N fertilization (Figure 2E), where a higher maize plant density increased N accumulation significantly for both intercropped and sole maize during the entire growth period except for the period before DAS 45. There was a slow N accumulation period before DAS 45, followed by a rapid accumulation to DAS 115, and then leveled off or declined to maturity. Averaged across the entire growth period, intercropped maize increased N accumulation by 17.7% compared to the monoculture maize and the increase was most significant from DAS 115 to 155 (Figure 2F). During the later growth period (after DAS 115), N fertilization significantly promoted N accumulation in both intercropped and sole maize.

### C and N Translocation in Pea and Maize Plants

#### C Translocation and Translocation Rate

For pea, the N × D interaction was not significant for the translocation-related variables (Table 1). However, N rate had a significant effect on C translocation and translocation rate in stem. On average, intercropped pea decreased the amount of C translocated to the grain from pea stem by 42.2% and C translocation rate by 36.9% with the N1 treatment compared with the N0 treatment. Unlike intercropped pea, monoculture pea increased C translocation amount in stem by 44% with the N1 treatment, while N rate did not affect C translocation or the translocation rate in either stem or leaf.

**TABLE 1:** Carbon accumulation, translocation and translocation rate in the stem, and leaf of intercropped pea and sole-planted pea under different N-fertilizer rates and plant densities in an Oasis irrigation region.

**N^a^ level**	**Plant density^b^**	**C accumulation (kg ha^–1^)^d^**				**C translocation (kg ha^–1^)^d^**		**C translocation rate (%)^d^**	
				
		**Highest value**		**At maturity**					
					
		**Stem**	**Leaf**	**Stem**	**Leaf**	**Stem**	**Leaf**	**Stem**	**Leaf**
Intercropped pea									
0	D1	872.7	1099.1	638.1	597.2	234.6	501.9	26.3	35.6
	D2	879.3	1234.8	559.6	481.2	319.7	753.6	34.7	43.1
	D3	807.9	873.2	566.5	481.4	241.4	391.8	29.0	33.8
135	D1	789.8	1023.3	549.6	737.9	240.2	285.4	27.0	29.0
	D2	848.9	1119.7	717.9	626.8	131.0	492.9	15.5	40.7
	D3	618.0	903.2	529.3	543.7	88.7	359.4	14.3	35.8
Sole pea									
0	–	685.6	960.1	492.1	450.4	193.5	509.7	33.4	53.3
135	–	705.4	1093.2	426.8	572.0	278.6	521.2	33.7	42.1
LSD (0.05)^c^		136	239	152	67	96	246	11.6	7.6
Significance (*p*-value)									
N level (N)		0.050	0.584	0.801	0.000	0.014	0.114	0.011	0.459
Plant density (D)		0.049	0.082	0.251	0.001	0.305	0.119	0.541	0.062
N × D		0.389	0.812	0.077	0.333	0.139	0.623	0.113	0.536

*^*a*^N-fertilizer at 0 and 450 kg N ha^–1^ for maize, 135 kg N ha^–1^ for pea. ^*b*^D1, D2, and D3 means maize plant density at 42,600, 49,700, and 56,900 plants ha^–1^, respectively. ^*c*^LSD was for the treatments in the same column. ^*d*^Each value was a 3-year mean, as there was no significant year by treatment interaction revealed in the ANOVA.*

For maize crops, there was a lack of significant effect from either N rate or plant density, and there were no any two-way or three-way interactions for the C translocation-related variables (Table 2). However, the amount of C accumulated in plant tissues varied significantly with N rate and plant density. On average, intercropped maize with N fertilizer (N1) increased stem C accumulation at the peak stage by 16.1% and leaf C accumulation by 11.7%, relative no-N treatment; similarly, monoculture maize increased the two values by 12.7 and 12.2%, respectively. At maturity, the N1 treatment increased intercropped maize C accumulation by 16.3% in stem and 11.4% in leaves; similarly, the N1 treatment increased monoculture maize C by 18.3% in stem and 12.2% in leaf compared with N0 treatment. Averaged across the two cropping patterns, maize with high plant density (D3) increased stem C accumulation by 30% and leaf C accumulation by 23% compared to maize with low plant density (D1).

**TABLE 2:** Carbon accumulation, translocation and translocation rate in the stem, and leaf of intercropped maize and sole-planted maize under different N-fertilizer rates and plant densities in an Oasis irrigation region.

**N level^a^**	**Plant density^b^**	**C accumulation (kg ha^–1^)^d^**				**C translocation (kg ha^–1^)^d^**		**C translocation rate (%)^d^**	
				
		**Highest value**		**At maturity**					
					
		**Stem**	**Leaf**	**Stem**	**Leaf**	**Stem**	**Leaf**	**Stem**	**Leaf**
Intercropped maize									
0	D1	668.1	874.6	522.5	542.3	145.5	332.4	20.5	30.1
	D2	748.0	1103.6	586.4	596.1	161.6	507.6	20.4	31.8
	D3	805.9	1175.4	593.7	602.0	212.2	573.4	22.6	39.0
450	D1	709.5	1104.7	583.8	607.4	125.8	497.3	16.1	38.0
	D2	864.0	1112.4	646.8	670.7	217.2	441.7	25.3	34.1
	D3	1007.3	1306.3	749.8	661.5	257.5	644.8	23.4	44.6
Sole maize									
0	D1	692.2	932.7	494.3	599.4	197.9	333.3	23.5	30.8
	D2	742.5	1118.3	591.7	658.4	150.7	459.9	15.4	30.2
	D3	946.2	1429.2	736.6	774.4	209.7	654.8	22.0	42.0
450	D1	837.8	1278.9	633.5	627.2	204.2	651.7	22.0	43.2
	D2	850.1	1375.1	682.8	766.2	167.3	608.9	17.6	36.6
	D3	995.4	1251.9	840.7	887.7	154.7	364.2	14.7	27.6
LSD (0.05)^c^		88	123	85	101	87	142	8.7	8.6
Significance (*p*-value)									
Cropping system (C)		0.221	0.023	0.154	0.014	0.866	0.824	0.533	0.722
N level (N)		0.004	0.012	0.006	0.074	0.814	0.313	0.800	0.336
Plant density (D)		0.000	0.002	0.001	0.032	0.595	0.325	0.968	0.483
C × D		0.556	0.948	0.378	0.252	0.347	0.381	0.402	0.482
N × D		0.932	0.050	0.805	0.882	0.849	0.055	0.668	0.235
C × N × D		0.335	0.070	0.730	0.888	0.758	0.091	0.812	0.267

*^*a*^N-fertilizer at 0 and 450 kg N ha^–1^ for maize, 135 kg N ha^–1^ for pea. ^*b*^D1, D2, and D3 means maize plant density at 42,600, 49,700, and 56,900 plants ha^–1^, respectively. ^*c*^LSD was for the treatments in the same column. ^*d*^Each value was a 3-year mean, as there was no significant year by treatment interaction revealed in the ANOVA.*

#### N Translocation and Translocation Rate

There were significant differences in N accumulation and translocation between intercrops and monoculture crops (Tables 3, 4). For pea, intercropping promoted N translocation rate in stem and leaf significantly, compared to monoculture pea (Table 3). The N translocation rate in intercropped pea leaf was 12% higher than that in monoculture pea leaf under N1 treatment. Moreover, increasing plant density promoted N translocation rate in intercropped pea leaf. On average, N translocation rate in intercropped pea leaf with high maize plant density (D3) was 19.4 and 3.8% higher than that with low (D1) and medium (D2) plant density with N application, and was 102.8 and 20.37% higher without N application, respectively. There was no significant N × D interaction in affecting N translocation rate in pea.

**TABLE 3:** Nitrogen accumulation, translocation and translocation rate in the stem and leaf of intercropped pea, and sole-planted pea under different N-fertilizer levels and plant densities.

**N level^a^**	**Plant density^b^**	**N accumulation (kg ha^–1^)^d^**				**N translocation (kg ha^–1^)^d^**		**N translocation rate (%)^d^**	
				
		**Highest value**		**At maturity**					
					
		**Stem**	**Leaf**	**Stem**	**Leaf**	**Stem**	**Leaf**	**Stem**	**Leaf**
Intercropped pea									
0	D1	51.2	33.0	31.4	18.7	19.9	14.3	37.2	32.2
	D2	51.0	31.2	30.3	13.6	20.7	17.6	39.2	52.0
	D3	43.2	28.4	25.3	9.5	17.8	18.9	42.1	65.3
135	D1	40.5	28.8	26.1	16.4	14.4	12.4	35.4	41.3
	D2	42.7	30.0	25.1	15.0	17.6	15.0	39.7	47.5
	D3	39.4	26.0	19.4	12.2	20.0	13.8	45.3	49.3
Sole pea									
0	–	32.1	18.7	19.6	8.3	12.4	10.4	37.8	51.9
135	–	37.1	21.5	19.7	11.7	17.4	9.8	44.8	41.1
LSD (0.05)^c^		6.9	5.9	5.5	3.8	6.0	7.2	11.7	13.3
Significance (*p*-value)									
N level (N)		0.045	0.391	0.065	0.758	0.468	0.382	0.900	0.537
Plant density (D)		0.387	0.529	0.160	0.039	0.821	0.727	0.535	0.044
N × D		0.707	0.911	0.992	0.545	0.547	0.928	0.931	0.262

*^*a*^N-fertilizer at 0 and 450 kg N ha^–1^ for maize, 135 kg N ha^–1^ for pea. ^*b*^D1, D2, and D3 means maize plant density at 42,600, 49,700, and 56,900 plants ha^–1^, respectively. ^*c*^LSD was for the treatments in the same column. ^*d*^Each value was a 3-year mean, as there was no significant year by treatment interaction revealed in the ANOVA.*

**TABLE 4:** Nitrogen accumulation, translocation and translocation rate in the stem, and leaf of intercropped maize and sole-planted maize under different N-fertilizer levels and plant densities.

**N level^a^**	**Plant density^b^**	**N accumulation (kg ha^–1^)^d^**				**N translocation (kg ha^–1^)^d^**		**N translocation rate (%)^d^**	
				
		**Highest value**		**At maturity**					
					
		**Stem**	**Leaf**	**Stem**	**Leaf**	**Stem**	**Leaf**	**Stem**	**Leaf**
Intercropped maize									
0	D1	24.3	34.4	14.1	14.7	10.2	19.7	39.3	51.6
	D2	25.5	39.6	14.7	15.2	10.8	24.5	38.0	52.6
	D3	28.5	40.9	14.2	14.5	14.3	26.4	49.1	60.9
450	D1	28.5	46.6	17.7	18.5	10.7	28.1	36.4	56.1
	D2	38.2	49.3	19.7	20.9	18.4	28.4	47.0	53.9
	D3	45.6	59.2	23.1	20.4	22.5	38.8	47.7	62.8
Sole maize									
0	D1	26.6	36.8	13.2	16.7	13.4	20.1	43.3	49.2
	D2	29.2	44.9	15.4	17.4	13.7	27.5	42.0	53.5
	D3	33.4	51.7	19.0	19.3	14.4	32.4	40.8	59.9
450	D1	33.3	52.7	19.5	19.1	13.8	33.7	37.1	57.4
	D2	32.8	55.1	22.6	23.8	10.2	31.3	27.8	50.5
	D3	39.5	50.4	25.9	29.1	13.6	21.3	32.8	39.7
LSD (0.05)^c^		3.7	4.8	2.3	2.9	4.0	5.1	9.7	7.8
Significance (*p*-value)									
Cropping system (C)		0.634	0.070	0.035	0.005	0.413	0.966	0.157	0.152
N level (N)		0.000	0.000	0.000	0.000	0.204	0.018	0.314	0.700
Plant density (D)		0.000	0.008	0.002	0.051	0.109	0.237	0.663	0.687
C × D		0.395	0.604	0.318	0.139	0.143	0.149	0.329	0.255
N × D		0.246	0.469	0.414	0.245	0.711	0.127	0.969	0.145
C × N × D		0.146	0.040	0.525	0.621	0.328	0.019	0.532	0.237

*^*a*^N-fertilizer at 0 and 450 kg N ha^–1^ for maize, 135 kg N ha^–1^ for pea. ^*b*^D1, D2, and D3 means maize plant density at 42,600, 49,700, and 56,900 plants ha^–1^, respectively. ^*c*^LSD was for the treatments in the same column. ^*d*^Each value was a 3-year mean, as there was no significant year by treatment interaction revealed in the ANOVA.*

For maize crops, N and D had significant effects on the amounts of N accumulated in stem and leaves (Table 4). Intercropped maize with the N1 treatment increased the N accumulation in stem by 43.4% and in leaves by 35.0% at the peak stage and the increases were 40.7% in stem and 34.7% in leaves at maturity, compared to the no-N treatment; similarly, monoculture maize with the N1 treatment increased N accumulation by 18.4, 18.6, 42.9, and 34.8%, respectively, in the two plant parts and at the two stages, compared with the no-N treatment. Plant density affected maize N accumulation significantly (Table 4). Averaged across all the treatments, the D3 maize plants increased the amount of N accumulation in stem by 30.4% and in leaf by 18.6% compared to the D1 maize at the peak stage, and by 27.4 and 20.7%, respectively, at the maturity stage (Table 4).

### Crop Growth Rate and Compensation Effect of Intercropped Maize After Pea Harvest

#### Crop Growth Rate of Intercropped Maize

After the harvest of the early-maturing intercropped pea, intercropped maize increased CGR significantly compared to monoculture maize (Table 5). On average, intercropped maize had 70.3% greater CGR at the pre-tasseling to silking stage, 159.8% greater at the silking – grain filling stage, and 154.8% greater at the hard dough stage, compared to monoculture maize. N rate and plant density individually had a significant effect on CGR. On average, fertilized maize increased CGR by 26.5, 3.4, 28.2, and 16.3% than the no-fertilized maize, at pre-tasseling to silking, silking to grain filling, grain filling to dough, and dough to full filling stages, respectively; the D3 maize plants increased CGR by 50.5, 36.6, 13.0, and 8.8% than D1 maize during the four growth periods.

**TABLE 5:** Crop growth rate (CGR, kg ha^–1^ d^–1^) of sole or intercropped maize after pea harvest in the four recovery stages as affected by N-fertilizer level and plant density.

**N level^a^**	**Plant density^b^**	**Pre-tasseling → silking**		**Silking → grain filling**		**Grain filling → dough stage**		**Dough stage → full maturity**	
					
		**Sole crop^d^**	**Intercrop^d^**	**Sole crop^d^**	**Intercrop^d^**	**Sole crop^d^**	**Intercrop^d^**	**Sole crop^d^**	**Intercrop^d^**
0	D1	71.5	80.7	61.4	157.4	33.9	148.0	9.4	11.0
	D2	80.1	149.5	71.1	213.1	51.1	86.7	12.0	11.9
	D3	103.2	162.5	74.8	220.4	51.9	108.8	8.4	5.0
450	D1	87.8	152.0	75.2	162.3	51.2	116.0	8.9	11.8
	D2	91.6	163.3	80.2	180.1	60.8	154.1	6.6	8.5
	D3	108.4	216.0	88.6	239.3	60.1	173.8	21.7	9.6
LSD (0.05)^c^		13.7	28.1	13.4	17.6	16.8	14.2	15.6	4.0
Significance (*p*-value)									
Cropping system (C)		0.034		0.000		0.000		0.053	
N level (N)		0.001		0.018		0.018		0.000	
Plant density (D)		0.000		0.000		0.000		0.011	
C × D		0.559		0.040		0.040		0.036	
N × D		0.754		0.933		0.933		0.581	
C × N × D		0.311		0.239		0.239		0.774	

*^*a*^N-fertilizer at 0 and 450 kg N ha^–1^ for maize, 135 kg N ha^–1^ for pea. ^*b*^D1, D2 and D3 means maize plant density at 42,600, 49,700, and 56,900 plants ha^–1^, respectively. ^*c*^LSD was for the treatments in the same column. ^*d*^Each value was a 3-year mean, as there was no significant year by treatment interaction revealed in the ANOVA.*

#### Compensatory Effect of Intercropped Maize

**FIGURE 3:**
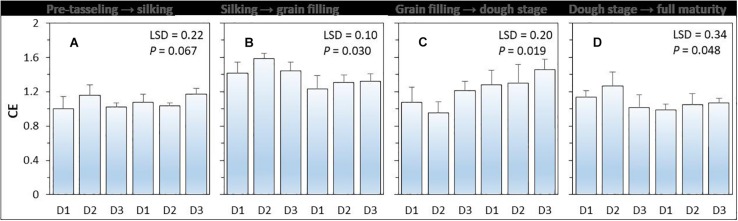
The compensation effect (CE) of intercropped maize during the four growth periods **(A–D)** under different N-fertilizer levels and plant densities. N0 represents N fertilizer at 0 kg N ha^–1^ and N1 represents N fertilizer rate at 450 kg N ha^–1^ for maize and 135 kg N ha^–1^ for pea. D1, D2, and D3 means maize plant density at low, medium, and high for the intercropped maize. The error bars are standard errors of the means (*n* = 3).

Compensatory effect (i.e., CE) had a value higher than 1 (Figure 3). There was a significant (*P* = 0.048) effect of planting density on CE, but the effect of N-fertilizer (*P* = 0.396) and planting density × N fertilizer interaction were not significant (*P* = 0.396). On average, the CE of intercropped maize was highest during the silking to grain filling period without N application and was highest form grain filling to R4 dough stage (Ransom, 2013) with N application. Under N-applied system, increased maize plant density increased the CE of intercropped maize. The CE of intercropped maize with high density was 6.0% higher than that with medium density, and 22.5% higher than that with low plant density.

### Grain Yields, Harvest Index and Land Equivalent Ratio

#### Grain Yields and Harvest Index of Pea

On average, the GYs of both intercropped pea and monoculture pea were higher under N-applied system than under no-N system, and the total yield increased with the increase of maize plant density in the intercropping system (Table 6). However, there was a significant year × treatment interaction in affecting GY. Compared to no-N treatments, N application increased the GY of intercropped pea by 18.9, 14.2, and 14.4% under low, medium, and high plant densities in 2012 and by 22.7, 15.9, and 11.5% in 2013; however, in 2014 N application increased intercropped pea yield by 13.2 % under high maize density and the N rate effects were not significant under medium and low densities. HI of the pea under intercropping systems was 5.7, 24.7, and 39.3% higher than that under sole cropping systems without N application in 2012, 2013, and 2014; and 10.4% and 4% higher with N application in 2013 and 2014, respectively. Under sole cropping systems, HI of pea with N application was 28.1, 24.1, and 22.4% higher than that without N application in 2012, 2013, and 2014. Under intercropping systems, HI of pea with N application was 6.4% and 9.9% higher than that without N application in 2012 and 2013, respectively.

**TABLE 6:** Grain yield (GY) and harvest index (HI) of pea under intercropping and sole cropping systems as affected by N-fertilizer level and plant density.

**N level^a^**	**Plant density^b^**	**Grain yield (kg ha^–1^)**			**Harvest index (%)**		
			
		**2012**	**2013**	**2014**	**2012**	**2013**	**2014**
Intercropping							
0	D1	1707	2172	2003	40.1	48.3	40.5
	D2	1713	2278	2058	38.7	54.6	50.6
	D3	1700	1948	1890	39.8	49.0	52.7
135	D1	2030	2665	2268	41.9	54.5	39.5
	D2	1957	2640	2154	40.9	55.7	44.1
	D3	1945	2172	1952	43.4	56.7	47.8
Sole cropping							
0	–	3146	3380	3298	37.4	40.6	34.4
135	–	4603	4753	4682	47.9	50.4	42.1
LSD (0.05)^c^		59	133	66	1.8	3.3	2.4
Significance (*p*-value)							
N level (N)		0.000	0.000	0.000	0.014	0.001	0.000
Plant density (D)		0.444	0.000	0.000	0.283	0.057	0.000
N × D		0.471	0.181	0.025	0.679	0.081	0.041

*^*a*^N-fertilizer at 0 and 450 kg N ha^–1^ for maize, 135 kg N ha^–1^ for pea. ^*b*^D1, D2, and D3 means maize plant density at 42,600, 49,700, and 56,900 plants ha^–1^, respectively. ^*c*^LSD was for the treatments in the same column.*

#### Grain Yields and Harvest Index of Maize

The GY of maize increased with the increase of maize plant density in all three study years. The GYs of the intercropped maize were higher than that of monoculture maize (Table 7). Without N application, the GY of maize under intercropping systems was 25.5, 25.8, and 30.7% higher than that of sole maize, in 2012, 2013, and 2014, respectively. With N application, the GY of maize under intercropping systems was 17.9, 23.2, and 21.7% higher than that of sole maize in 2012, 2013, and 2014, respectively. In both N-applied and no-N system, HI of monoculture maize was higher than that of intercropped maize.

**TABLE 7:** Grain yield (kg ha^–1^) and HI (%) of maize under intercropping and sole cropping systems as affected by N-fertilizer level and plant density.

**N level^a^**	**Plant density^b^**	**Grain yield**			**Harvest index**		
			
		**2012**	**2013**	**2014**	**2012**	**2013**	**2014**
Intercropping							
0	D1	9131	9289	9257	55.4	55.5	55.5
	D2	10512	10404	10505	53.1	53.9	53.6
	D3	11312	11811	11608	47.4	52.3	49.9
450	D1	10556	10411	10530	51.3	50.1	50.8
	D2	12375	12052	12261	51.9	50.0	51.0
	D3	12485	12220	12433	47.8	47.0	52.3
Sole cropping							
0	D1	6797	7327	6209	65.4	64.9	57.0
	D2	8203	8350	8223	66.8	65.9	57.3
	D3	9672	9358	9562	70.7	65.4	58.4
450	D1	8633	8174	8451	64.6	61.0	57.3
	D2	10347	9232	9536	66.7	56.7	50.3
	D3	11055	10746	10947	59.3	55.1	57.2
LSD (0.05)^c^		137	247	121	1.5	2.1	1.3
Significance (*p*-value)							
Cropping system (C)		0.000	0.000	0.000	0.000	0.000	0.000
N level (N)		0.000	0.000	0.000	0.000	0.000	0.000
Plant density (D)		0.000	0.000	0.000	0.000	0.023	0.011
C × D		0.000	0.001	0.000	0.003	0.801	0.002
N × D		0.001	0.776	0.000	0.011	0.282	0.001
C × N × D		0.356	0.034	0.000	0.000	0.182	0.002

*^*a*^N-fertilizer at 0 and 450 kg N ha^–1^ for maize, 135 kg N ha^–1^ for pea. ^*b*^D1, D2, and D3 means maize plant density at 42,600, 49,700, and 56,900 plants ha^–1^, respectively. ^*c*^LSD was for the treatments in the same column.*

#### Land Equivalent Ratio of Various Intercropping Systems

**FIGURE 4:**
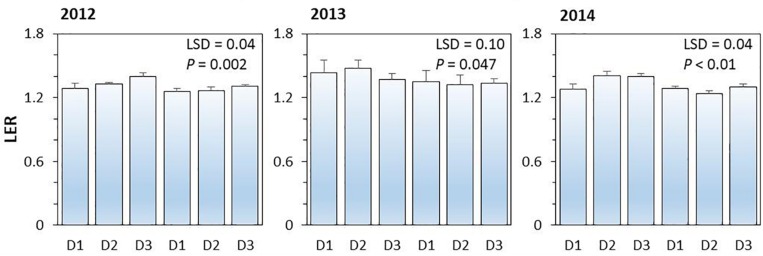
The land equivalent ratio (LER) of pea-maize intercropping systems under different N-fertilizer levels and plant densities in 2012, 2013, and 2014. N0 represents N fertilizer at 0 kg N ha^–1^ and N1 represents N fertilizer rate at 450 kg N ha^–1^ for maize and 135 kg N ha^–1^ for pea. D1, D2, and D3 means maize plant density at low, medium, and high for the intercropped maize. The error bars are standard errors of the means (*n* = 3).

On average, land equivalent ratio (i.e., LER) of the intercropping systems ranged from 1.26 to 1.40 in 2012, from 1.32 to 1.48 in 2013, and from 1.24 to 1.41 in 2014; the higher than 1 in LER indicated the yield advantages of intercropping over the corresponding monoculture (Figure 4). The effect of N-fertilizer on LER was significant (*P* < 0.001), but planting density effect (*P* = 0.254) and planting density × N fertilizer interaction were not significant (*P* = 0.114). N rate had a negative effect on LER; the average LER of intercropping systems with N application was 4.7, 6.8, and 6.8% lower than that without N application in 2012, 2013, and 2014, respectively. The increased value of LER without N application was due to the intercropped maize and intercropped pea produced more yields than the corresponding monoculture plants under no-N conditions.

## Discussion

Agriculture today faces significant challenges with changing climate (Smirnov et al., 2016), unpredictable weather patterns (Stott, 2016), and threats from various biotic stresses (Liu et al., 2010; Tao et al., 2011). These challenges decrease the agroecosystem productivity (Mondal et al., 2016) and profitability (Lin et al., 2008), and threat the long-term sustainability (Lin et al., 2008; Tao et al., 2011). Also, securing foods for the growing population on the planet brings in another challenge to agriculture. Therefore, it is imperative to develop strategies that enable to increase crop yields on the existing farmland substantially (Mueller et al., 2012; Zhang W. et al., 2016).

Numerous studies have shown that intercropping has substantial advantages in crop yield and many other benefits than monoculture cropping; this has been reported worldwide, including those in northwest China (Mu et al., 2013; Chen et al., 2014a; Hu et al., 2016), India (Nelson et al., 2018), France (Maxin et al., 2017; Kaci et al., 2018), Germany (Gronle et al., 2014; Munz et al., 2014), and the North America (Franco et al., 2018; Simpson et al., 2018). However, the published studies have rarely determined the processes of C and N accumulation in vegetative tissues and the remobilization capacity of the photosynthate to the grain sinks under intercropping systems. There is a huge knowledge gap how the C and N accumulation and translocation may be related to fertilizer application and plant densities of the intercrops. This gap needs to be filled to better use the benefits associated with intercropping. In the present study, we quantified the C and N accumulation in various plant tissues and determined the rate of photosynthate translocation from vegetative tissues to the grain sinks in response to fertilizer application and plant densities.

We found that intercropped maize and pea had significant advantages on C and N accumulation in plant tissues compared with corresponding monoculture crops, and these advantages were further enhanced with increased N fertilization and maize plant densities. High maize plant density increased the total C and N accumulation in the vegetative tissues of intercrops during the vegetative period. Increased maize plant density also increased the rates of C and N remobilization from both intercropped maize and pea to the grain sinks during the reproductive period. Our results indicate that the C and N accumulation potential in the intercrops can be excavated by properly increasing maize plant density. A simple agronomic practice (i.e., managing plant density) can lay an important foundation of photosynthetic materials in the plants during the late reproductive period. Previous studies on monoculture maize show that increasing maize plant density can increase maize GY and nitrogen use efficiency through the enhancement of canopy light absorption (Mohan Kumar et al., 2015) and the promotion of dry matter accumulation during the post-silking period (Zhou et al., 2019). However, the plant density effect can vary largely with genotype (Mastrodomenico et al., 2018), soil fertility (Zhou et al., 2019), water availability (Nyakudya and Stroosnijder, 2015), and the interaction between genotype × density interaction (Haegele et al., 2014). In the present study, we add a significant value to the scientific literature – increasing maize plant density increased C and N remobilization from the vegetative tissues to the grain sinks which helps offset the disadvantageous growth of intercropped maize plants inhibited during the earlier part of the co-growth period.

We also found that fertilizer application had a significant impact on C and N accumulation and translocation in plant tissues. Increased N fertilizer rate significantly increased C and N accumulation in intercropped maize across the three plant density treatments evaluated in the study. Unlike the intercropped maize, the C and N accumulation in intercropped pea gradually increased during the whole growth period until the mature stage, and at maturity, the rate of C and N accumulation decreased in the pea plant. The later-stage decreases were largely due to the intercropped maize that increased the competitive ability that inhibited the growth of the pea. We also observed that the intercropped pea plants withered nearly 2 months before maize maturation with a large proportion of the leaves fell to the ground at maturity; this may provide certain degree of nitrogen transfer from the mature pea to the vigorously growing maize plants. Although we did not quantify the amount of N that might be transformed from the falling leaves of pea plants in the present study, we provide new evidence that the potential N transfer by intercropped pea may contribute to the compensatory effect reported by other researchers (Chen et al., 2014a; Yin et al., 2017; Kaci et al., 2018).

In intercropping systems, interspecific interaction often occurs between the intercrops because of the mutual requirements for the same space and resources (Xu et al., 2010; Xia et al., 2013). Interspecific competition may promote the use of different resources in the soil, as the different crops use a given resource at different times or spaces (Li et al., 2014). Interspecific competition is bound to weaken the growth of late-maturing crops (i.e., the weaker competitive crops) during the co-growth period (Li et al., 2001a). Therefore, the yield advantage of intercropping is partly due to the compensation effect (CE) of the long-season crop whose growth is impaired during the co-growth period. In the intercropping pattern, after the harvest of early-maturing crops, the late-maturing crops could form the compensatory effect of time and space. In terms of compensatory effect on time, the growth period of the late-maturing crops can be extended and leaf area index can be increased (Li et al., 2001a, 2001b). In terms of CE on space, the late-maturing crop can root to the underground space that is occupied by the early-maturing crops after early-maturing crops are harvested (Wang et al., 2018); these effects expand the scope of nutrient uptake (Li et al., 2014) and water absorption (Chen et al., 2018), so that the growth rate of the late-maturing crop is accelerated and dry matter accumulation is increased, consequently, the yield of late-maturing crops can be close to or more than that produced by the corresponding monoculture crops (Chen et al., 2018; Martin-Guay et al., 2018; Qian et al., 2018; Wang et al., 2018; Xiao et al., 2018).

## Conclusion

Pea-maize intercropping had a greater advantage over the corresponding sole cropping and such effect was greatest under the high maize plant density. Increased maize plant density enhanced the compensatory effect of the intercropped maize after the harvest of the early-maturing pea, as the high maize density primarily promoted C and N accumulation in the vegetative tissues and in some cases enhanced C and N translocation from the vegetative tissues to the grain sinks. Meanwhile, the proper plant density in combination with N management significantly increased CGR of the intercropped maize, thus increasing the GY of the intercropped maize compared with the monoculture maize. The proper plant density with N management provided an effective means of enhancing the compensation of the impaired growth of intercropped maize through the improved C and N accumulation and translocation after pea harvest. Our results demonstrated that the proper plant density with N management played a significant role in promoting C and N accumulation and translocation, enhancing the compensatory effect of the late-maturing maize, and increasing the crop productivity in the arid oasis areas.

## Author Contributions

QC conceived and designed the experiments. YZ, ZF, FH, WY, CZ, and AY performed the data collection. YZ and ZF analyzed the data and interpreted the results. YZ wrote the manuscript. All authors contributed to the manuscript enhancement and finalization, read, and approved the submitted version.

## Conflict of Interest Statement

The authors declare that the research was conducted in the absence of any commercial or financial relationships that could be construed as a potential conflict of interest.
